# T cells expressing NKG2D chimeric antigen receptors efficiently eliminate glioblastoma and cancer stem cells

**DOI:** 10.1186/s40425-019-0642-9

**Published:** 2019-07-09

**Authors:** Dong Yang, Bin Sun, Hongjiu Dai, Wenxuan Li, Lan Shi, Peixian Zhang, Shirong Li, Xudong Zhao

**Affiliations:** 1 Key Laboratory of Animal Models and Human Disease Mechanisms of Chinese Academy of Sciences/Key Laboratory of Bioactive Peptides of Yunnan Province, Kunming Institute of Zoology, Chinese Academy of Sciences, 32 East Jiaochang Road, Kunming, 650223 Yunnan China; 2 Nanjing Kaedi Biotech Co. Ltd, 18 Zhilan Road, Nanjing, 211100 Jiangsu China; 3 Laboratory of tumor animal models and anti-aging, State Key Laboratory of Biotherapy and Cancer Center, West China Hospital, Sichuan University, Chengdu, 610041 Sichuan China; 4 College of Life Sciences, Sichuan University, Chengdu, 610064 Sichuan China; 5 Oncology Department of Yanan Hospital, Kunming, 650051 China; 6 Center for Excellence in Animal Evolution and Genetics, Chinese Academy of Sciences, Kunming, 650223 China

**Keywords:** Chimeric antigen receptor, Glioblastoma, Cancer stem cell, NKG2D

## Abstract

**Background:**

Traditional therapies fail to cure most glioblastoma patients and the 5-year survival rate is less than 10%, highlighting need for new therapeutic approaches. The natural killer group 2 member D ligands (NKG2DLs) are highly expressed in glioblastomas and are considered promising targets for chimeric antigen receptor (CAR) T-cell therapy. The aim of this study was to investigate the effect of NKG2D-expressing CAR-T cells on glioblastomas and glioblastoma stem cells.

**Methods:**

The expression of NKG2DLs was analyzed by flow cytometry and immunohistochemistry. NKG2D-BBz CAR, containing the extracellular domain of NKG2D, was constructed and delivered into T cells by lentiviral particles. In vitro cytotoxicity of the CAR-T cells was assessed by flow cytometry. Release of cytokine, perforin and granzyme B was quantified using enzyme-linked immunosorbent assay kits. The therapeutic efficacy of NKG2D-BBz CAR-T cells in vivo was evaluated using subcutaneous tumor models. The safety of the CAR was analyzed by investigating the effects on proliferation, apoptosis, and karyotype.

**Results:**

Our data confirmed the high expression of NKG2DLs in human glioblastoma cells, cancer stem cells, and tumor samples. Further, the NKG2D-BBz CAR-T cells efficiently lysed glioblastoma cells and cancer stem cells in vitro and produced high levels of cytokines, perforin, and granzyme B. The CAR-T cells markedly eliminated xenograft tumors in vivo and did not exhibit significant treatment-related toxicity in the treated mice. The CAR expression also did not exert any obvious effects on cell proliferation, apoptosis, and genomic stability.

**Conclusion:**

Our findings demonstrated that NKG2D CAR-T cells targeted glioblastoma cells and cancer stem cells in an NKG2D-dependent manner, supporting the use of CAR-T therapy in glioblastoma therapeutic strategies.

**Electronic supplementary material:**

The online version of this article (10.1186/s40425-019-0642-9) contains supplementary material, which is available to authorized users.

## Background

Glioblastoma is the most aggressive cancer of the central nervous system. Over the past few decades, although some progress has been made in traditional treatment options, consisting of surgery, radiotherapy, and chemotherapy, the 5-year survival rate of glioblastoma patients is still less than 10%, due to the biological complexity of brain tumors [1]. The blood-brain barrier, a highly selective semipermeable border that separates the circulating blood from the brain and extracellular fluid in the central nervous system, prevents most cancer therapeutics from entering the brain parenchyma [2]. Only three new treatments, with minor improvement in prognosis, have been approved by the FDA for glioblastoma since 2005, including temozolomide, bevacizumab, and tumor-treating fields [3]. Therefore, there is an urgent need to develop new treatment strategies for glioblastoma.

Recently, immunotherapy using engineered T cells expressing chimeric antigen receptors (CAR) showed remarkable curative effects in multiple tumor types, especially, in the case of hematologic malignancies [4]. CARs are artificial fusion proteins that include an extracellular antigen-binding domain, a transmembrane domain, and intracellular T-cell signaling domains (such as CD3ζ alone or in combination with one or more costimulatory components) [5]. It’s reported that T cells can cross the blood-brain barrier and infiltrate the brain by diffusion [6]. Thus, CAR-T cell-based immunotherapy for glioblastomas could overcome the difficulty of drug delivery. So far, CAR-T cells against IL-13Ra2, EGFRVIII, and HER2 have been tested in glioblastoma clinical treatment and have showed promising results [7–9]. Moreover, CAR-T cell immunotherapy targeting EphA2, MUC1, EGFR, PD-L1, and PDPN are currently undergoing clinical studies in glioblastoma, but the results have not yet been made public [10–12]. These reports indicate that CAR-T cell therapy could be a new treatment for glioblastoma patients. However, there are still some limitations that prevent the application of such therapies, such as the heterogeneity of glioblastoma and immunosuppression of tumor microenvironment. Thus, it is still necessary to develop new CARs for the treatment of glioblastoma. Natural killer group 2 member D (NKG2D) ligands, including two MHC class I-related chains and six UL16-binding proteins, were reported to be expressed at low levels in healthy human tissues and to be upregulated in most cancers [13]. NKG2D-expressing CARs were demonstrated to be effective against most cancer types, such as multiple myeloma, ovarian carcinoma, and lymphoma [14]. CAR-T cells expressing the full length murine NKG2D were also reported to remarkably prolong the survival of mice bearing intracranial xenograft of mouse glioma cells [15]. However, the exact role of NKG2D-expressing CARs in human glioblastoma remains to be elucidated.

The existence of cancer stem cells (CSCs) in glioblastoma is considered one of the major causes of the conventional therapy failure and cancer recurrence [16, 17]. Previous studies have shown that the targeted killing of CSCs could effectively inhibit glioblastoma tumorigenesis and prolong the survival of glioma-bearing mice [18]. Therefore, development of specific and effective therapeutic approaches to eliminate CSCs is urgently required. Due to the strong resistance to radiotherapy and chemotherapy, immunotherapy shows more promising potential to eradicate CSCs. Many surface markers of CSCs have been used as target antigens for immunotherapy in cancer treatment, such as CD133, CD90, ALDH, and EpCAM, and revealed encouraging anticancer activity [19]. Previous studies have proved that natural killer cells could efficiently target osteosarcoma stem cells in an NKG2D-NKG2DL dependent manner [20]. NKG2D ligand (NKG2DL) overexpression was reported in glioblastoma stem cells (GSCs), suggesting that NKG2D-expressing CAR-T cells may also have an effect on GSCs [21].

In this study, we constructed CAR-T cells expressing the extracellular domain of human NKG2D, as well as the 4-1BB and CD3ζ signaling domains, and investigated the efficacy of these NKG2D-expressing CAR-T cells against human glioblastoma cells and CSCs.

## Methods

### Cell culture

U-251 MG, T98G, U-87 MG and HTB185 cell lines were purchased from China Infrastructure of Cell Line Resources (Kunming, China). The cells were tested via short tandem repeat (STR) profiling in October 2018. The cells were cultured in DMEM containing 10% fetal bovine serum (Millipore, USA), 100 U/ml penicillin, and 100 mg/ml streptomycin (Life Technologies, USA). The GSC-3# cell line, isolated from primary glioblastoma tissues, was described previously [22]. U-251 MG and U-87 MG suspended cell spheres, named U251-CSC and U87-CSC, respectively, were established using serum-free neural stem cell medium composed of DMEM/F12, EGF (20 ng/ml, Life Technologies, USA), bFGF (20 ng/ml, Life Technologies, USA), and B27 (1x, Life Technologies, USA) [23, 24]. All cell lines were cultured in a humidified incubator with 5% CO_2_ at 37 °C.

### Vector design

The CD19 targeting domain and the extracellular domain of human NKG2D was synthesized (Idobio, China) and cloned into a CAR-encoding lentivirus backbone and named CD19-BBZ CAR and NKG2D-BBZ CAR, respectively, containing a CD8 hinge spacer and transmembrane domain, 4-1BB, and CD3ζ endo-domains. The CARs were cloned into the LentiGuide-Puro plasmid (Addgene, USA) and expressed under the control of an EF1a promoter.

### Lentiviral package

The lentiviral plasmids were co-transfected into HEK293T cells with the packaging plasmids psPAX2 and pCMV-VSVG (Addgene, USA) at a ratio of 10:8:5 as described before [25].

### CAR-T cell preparation

Blood collected from three healthy donors was used to isolated T cells respectively using the RosetteSep™ Human T Cell Enrichment Cocktail (STEMCELL, Canada) and the T cells were cultured in RPMI 1640 medium (Life Technologies, USA) containing 10% FBS (Life Technologies, USA) with 200 U/ml IL-2 (PeproTech, USA). T cells were activated by CD3/CD28 Dynabeads (Life Technologies, USA) according to the manufacturer’s instructions. After 48 h, lentiviral particles were added to the cultures at a multiplicity of infection (MOI) of 10 in the presence of polybrene at a final concentration of 8 μg/ml. The CAR-T cells were counted on alternate days and fresh medium was added to the cultures to maintain the cell density at 1 × 10^6^ cells/ml. Four days after T cells were infected with CAR lentivirus, total T cells were used for in vitro experiments. When the T cells were expanded in vitro for an additional 6 days, the cells were collected and used for the in vitro experiments.

### Cytotoxicity assay

The antitumor activity of CAR-T cells was evaluated using a Cell-Mediated Cytotoxicity Fluorometric Assay Kit (BioVision, USA). Briefly, carboxyfluorescein succinimidyl ester (CFSE)-stained T98G, U-251 MG, and U-87 MG glioblastoma cells (target cells) were seeded into 96-wells at a density of 4 × 10^4^ cells/well. Subsequently, non-transduced T cells (NTD), CD19-BBz CAR, or NKG2D-BBz CAR-T cells (effector cells) were added to each well to ensure an effector:target cell (E:T) ratio of 8:1, 4:1, 2:1, 1:1, or 0.5:1. After 20 h of co-culture, the tumor cells were collected, and dead cells were stained with 7- aminoactinomycin (AAD) and quantified by flow cytometry. The concentrations of perforin and granzyme B in the supernatant of co-culture system were measured using enzyme-linked immunosorbent assay kits (abcam, USA).

### Cytokine secretion assay

The effector cells (NTD T cells, CD19-BBz CAR, or NKG2D-BBz CAR-T cells) were co-cultured with target cells (U-251MG glioblastoma cells) for 16 h at an E:T ratio of 5:1 and the medium supernatant was assessed for the levels of cytokine secretion. The concentrations of IL-2, IL-10, TNF-α, and IFN-γ were measured using enzyme-linked immunosorbent assay kits (BD Biosciences, USA) according to the manufacturer’s instructions.

### Flow cytometry

Cells were harvested, washed twice with 1× PBS, and resuspended in cold PBS containing 2% FCS, 1% sodium azide (at a density of 1 × 10^6^ cells/ml). Subsequently, primary labelled antibodies were added into the cell suspension according to the manufacturer’s instructions and incubated for 1 h at 4 °C in the dark. Immediately after the incubation, the cells were washed thrice with ice cold PBS and flow cytometry was performed using a BD LSRFortessa cell analyzer (BD Biosciences, USA). The data was analyzed using the FlowJo analysis software package (TreeStar, USA). Details of all antibodies are provided in Additional file 1.

### Xenograft mouse model

NOD-*Prkdc*^*scid*^ *IL2rg*^*tm1*^/Bcgen (B-NDG) mice [26] were purchased from Jiangsu Biocytogen Co., Ltd. (Nantong, China). Five to six-week-old B-NDG mice were bred under specific pathogen-free conditions. Stable luciferase transfected U-251MG and U-87MG cells (at a density of 1 × 10^7^ cells/ml) were suspended in PBS containing 30% matrigel (BD Bioscience, USA) and 100 μl of the cell suspension subcutaneously injected into B-NDG mice. After 7 days, the mice were anaesthetized and imaged using IVIS system followed by intraperitoneal injection of 150 mg/kg D-Luciferin (BioVision, USA). When the mean tumor bioluminescence reached ~ 5 × 10^7^ photons/second, mice were treated with 100 μl of normal saline (NS) or NTD T cells, CD19-BBz CAR, or NKG2D-BBz CAR-T cells (1 × 10^8^ cells/ml) by intravenous injection. The bioluminescent signals were measured every week. The data were quantified using Living Image software (Caliper Life Science, USA). All animal protocols were approved by the Institutional Review Board at Kunming Institute of Zoology, Chinese Academy of Sciences.

### RNA isolation and real-time PCR

The total RNA was isolated from the cell lines as previously described [27]. Relative expression of CSCs markers was detected in T98G, U-251MG, and U-87MG suspended cell spheres using a SYBR Green qPCR Kit (Life Technologies, USA). For the persistence of CAR-T cells in vivo, 50 μl of venous blood was collected weekly from the orbital venous plexus of the treated mice and genomic DNA was isolated using a tissue DNA kit (Omega Bio-tek, USA). The DNA content of the human genome detected in the extracted genomic DNA using the GAPDH primer (human specific). 18S rRNA was used as an internal control. All the primers used in this study are shown in Additional file 2.

### EdU (5-ethynyl-2-deoxyuridine) incorporation assay

EdU incorporation assay was performed using a Click-iT EdU Alexa Fluor 488 Imaging Kit (Thermo Fisher Scientific, USA) according to the manufacturer’s instructions. EdU was added to the cell culture medium at a final concentration 10 μM and incubated for 1 h. Cells were fixed with 4% paraformaldehyde in PBS followed by permeabilization with 0.3% Triton X-100 in PBS. Thereafter, the cells were incubated with Click-iT reaction cocktail containing Alexa Fluor® azide for 30 min at room temperature. The EdU incorporation rate was analyzed by flow cytometry.

### Immunohistochemical staining

Immunohistochemical analysis was carried out as described previously [25].The tissue microarrays used to detect the expression of NKG2DLs were purchased from Wuhan Servicebio Technology Co., Ltd. (Wuhan, China; cat. no. Glc 1601). The expression of NKG2D ligands was semiquantitatively evaluated as follows: - (absence of staining), 1+ (mild staining), 2+ (moderate staining), 3+ (severe staining). Details of all primary antibodies are provided in Additional file 1.

### Statistical analysis

Experiments were repeated at least three times unless specified otherwise in the text. All statistical analyses were performed using GraphPad Prism 7.0 statistical software. The data were presented as mean ± standard deviation (SD). Statistical differences between two groups were analyzed using unpaired two-tailed Student’s *t*-tests. Statistical differences among three or more groups were analyzed by one-way ANOVA. Statistical significance was defined as ^*^P ≤ 0.05, ^**^P ≤ 0.01, ^***^P ≤ 0.001.

## Results

### NKG2DL expression in glioblastoma cell lines and patient tumor tissues

**Fig. 1:**
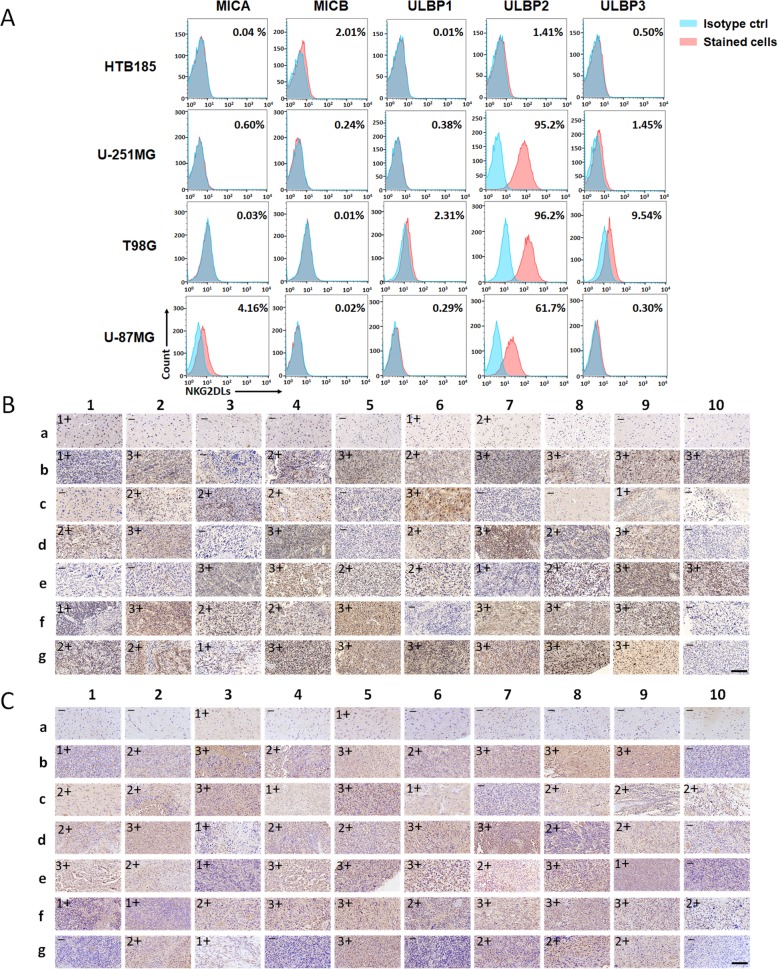
NKG2DL expression in glioblastoma cell lines and patient tumors. **a** Expression of NKG2D ligands for U-251MG, T98G, U-87MG, HTB185 cells. Percentage of positive cells is detailed in the histograms. **b**, **c** The major NKG2D ligand MICA and ULBP2 staining in a tissue microarray containing 60 glioblastoma tissues and 10 normal tissues. For each antibody, evaluate semiquantitatively the immunostaining, as follows: - (absence of staining), 1+ (mild staining), 2+ (moderate staining), 3+ (severe staining), scale bar = 300 μm

Cell surface expression of the main ligands for the NKG2D receptor (MICA, MICB, ULBP1, ULBP2, and ULBP3) on the glioblastoma cell lines, U-251MG, T98G, and U-87MG, was assessed by flow cytometry. As expected, all the tested glioblastoma cell lines expressed high levels of NKG2DLs, particularly ULBP2. NKG2DL-negative cell line, HTB185, was used as a negative control in the subsequent experiments (Fig. 1a).

In previous studies, expression of NKG2DLs in tumor samples was mainly detected at the mRNA level or at the protein level using few samples. Thus, we performed immunohistochemical analysis to determine and compare the expression of NKG2DLs in tissue microarrays (Servicebio Co., ltd, China) from 60 glioblastoma cancer tissues and 10 normal tissues. Our results showed that strong cell-surface expression (2+ and 3+) of MICA and ULBP2 was detected in about 68 and 72% of the cancer tissues, respectively. Meanwhile, very few cancer tissues expressed ULBP1 and ULBP3. On the other hand, only a few normal tissues exhibited slight expression of the NKG2DLs (Fig. 1b, c & Additional file 3: Figure S1, S2). Specifically, the expression of MICA in normal tissues was mainly localized in the cell nucleus (Fig. 1b).

### NKG2D-BBz CAR-T cells efficiently lyse glioblastoma cells in vitro

**Fig. 2:**
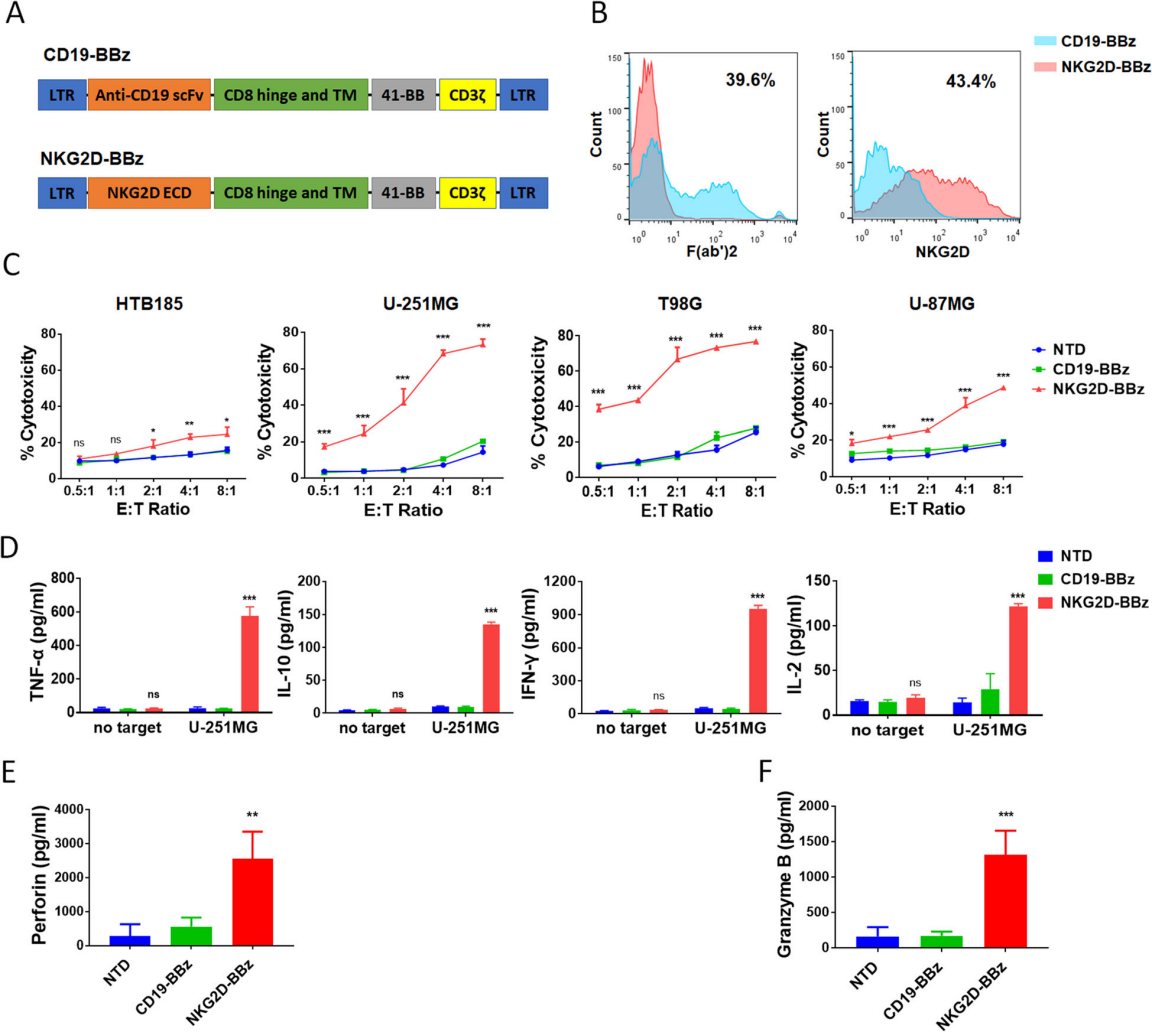
NKG2D-BBz CAR-T cells lyse glioblastoma cells efficiently in vitro*.* **a** The schematic diagram of CD19-BBz and NKG2D-BBz vectors. **b** Expression of CARs were confirmed by FACS with F(ab)2 antibody for CD19-BBz and NKG2D antibody for NKG2D-BBz. **c** The cytotoxicity of NTD, CD19-BBz and NKG2D-BBz T cells against the indicated cell lines at different effector to target (E: T) ratios. The results are presented as the mean volume ± SD, * *P* < 0.05, *** *P* < 0.001. **d** The levels of cytokines, released by NTD, CD19-BBz and NKG2D-BBz T cells, were measured by ELISA after 16 h co-culture incubation at E:T ratio of 5:1. The results are presented as the mean volume ± SD, *** *P* < 0.001, ns, not significant. **e**, **f** The levels of perforin and granzyme B in the supernatant of the co-culture system described above were measured by ELISA. The results are presented as the mean volume ± SD, ** *P* < 0.01, *** *P* < 0.001

The expression of NKG2DLs in glioblastoma cells and tumor tissues indicated that NKG2D-expressing CAR-T cells can be a potential therapy for glioblastoma. Thus, we constructed a NKG2D-CAR lentiviral vector (NKG2D-BBz) containing the NKG2D extracellular region, CD3ζ, and CD137 signaling domains (Fig. 2a). The CAR expressing CD19-scFv was used as the negative control (CD19-BBz).

T cells were acquired from healthy donors and confirmed by CD3 expression (Additional file 3: Figure S3). The cells were activated for 48 h using CD3/28 beads, and then transduced with NKG2D-BBz and CD19-BBz lentivirus. The expression of CARs was assessed by flow cytometry analysis on day 3. The percentage of T cells positive for NKG2D-BBz CAR was approximately 40% (Fig. 2b). To determine the cytotoxic capacity of NKG2D-BBz CAR-T cells against glioblastoma cells, we incubated T cells with T98G, U-251MG, and U-87MG glioblastoma cell lines at different E:T ratios of 8:1, 4:1, 2:1, 1:1, and 0.5:1. HTB185 cells served as the control cells. The results showed that NKG2D-BBz CAR-T cells efficiently lysed the glioblastoma cells, but not the HTB185 cells (Fig. 2c). U-87MG cells were the least sensitive to NKG2D BBz CAR-T cells due to the lowest level of NKG2DL expression among the three tested glioblastoma cell lines. On the other hand, NTD and CD19-BBz CAR-T cells showed very weak cytotoxicity (Fig. 2c).

Because cytokine secretion by CAR-T cells targeting cancer cells indicates the activation and specific cytotoxicity of T cells, we analyzed the levels of the classic cytokines, TNF-α, IL-10, INF-γ, and IL-2, to assess the cytokine profile when CAR-T cells were incubated with U-251MG cells. The concentrations of tested cytokines were significantly elevated in the supernatant of the NKG2D-BBz CAR-T co-culture system compared with those of U-251MG cells co-cultured with CD19-BBz CAR-T cells and NTD cells (*P* < 0.001, Fig. 2d). Furthermore, the increase in cytotoxic efficacy was consistent with increased concentrations of perforin and granzyme B in the supernatant of NKG2D-BBz CAR-T co-culture system (*P* < 0.001, Fig. 2e & f).

### NKG2D-BBz CAR-T cells exhibited potent cytotoxicity against GSCs

**Fig. 3:**
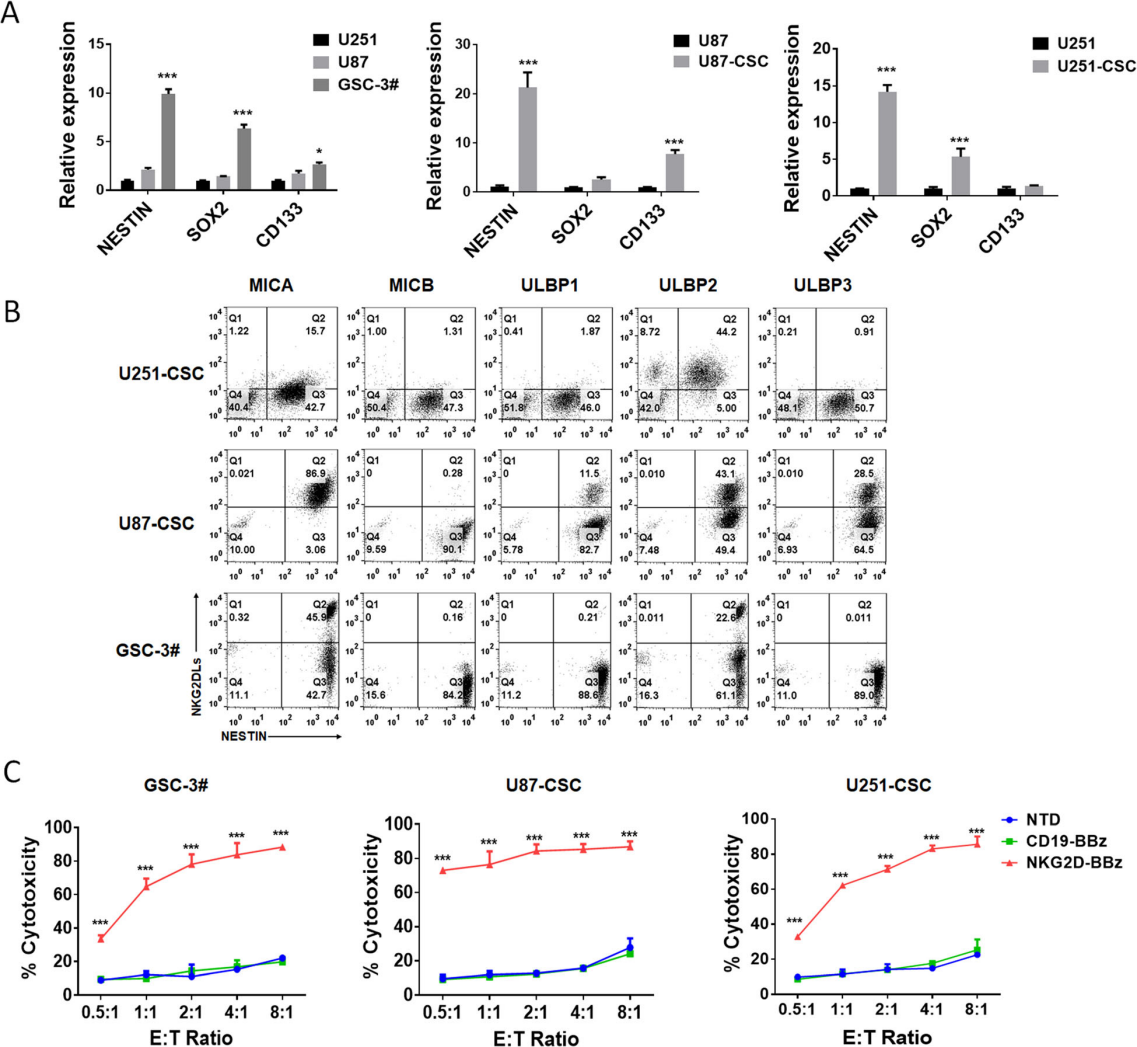
NKG2D-BBz CAR-T cells had a potent cytotoxicity targeting glioblastoma CSCs. **a** The expression of CSC markers SOX2, nestin, and CD133 was detected by realtime PCR. The results are presented as the mean volume ± SD, * *P* < 0.05, ** *P* < 0.01, *** *P* < 0.001. **b** The co-expression of the glioblastoma stem cell marker NESTIN and NKG2DLs in GSC-3#, U251-CSC, U87-CSC cells was assessed by flow cytometry. Percentage of positive cells is detailed in the histograms. **c** Cytotoxicity of NTD, CD19-BBz and NKG2D-BBz T cells against cancer stem cells at different effector to target (E: T) ratios. The results are presented as the mean volume ± SD, *** *P* < 0.001

To check if NKG2D-BBz CAR-T cells possess activity against GSCs, we generated suspended cell spheres from primary glioblastoma tissues, U-251MG cells, and U-87MG cells, and named GSC-3#, U251-CSC, U87-CSC, respectively (Additional file 3: Figure S4). The expression of GSC markers, such as NESTIN, SOX2, and CD133, was measured in the suspended cell spheres by real time PCR. Our results showed that NESTIN was significantly upregulated in all the tested spheres. In addition, the expression of SOX2 in GSC-3# and U251-CSC and the expression of CD133 in U87-CSC were also elevated (Fig. 3a). These data indicated that GSCs were enriched in these cell spheres. Next, the co-expression of the glioblastoma stem cell marker NESTIN and NKG2DLs (MICA, MICB, ULBP1, ULBP2, ULBP3) in these suspended cell spheres was assessed by flow cytometry. Strong cell-surface expression of MICA in GSC-3# and U87-CSC, and ULBP2 in U251-CSC and U87-CSC was detected (Fig. 3b). NKG2D-BBz CAR-T cells, but not CD19-BBz CAR-T cells and NTD cells, exhibited potent cytotoxicity and strong cytokine release, when they were incubated with the suspended cell spheres (Fig. 3c & Additional file 3: Figure S5).

### NKG2D-BBz CAR-T cells showed effective and persistent antitumor activity against xenografts formed by U-251MG cells in mice

**Fig. 4:**
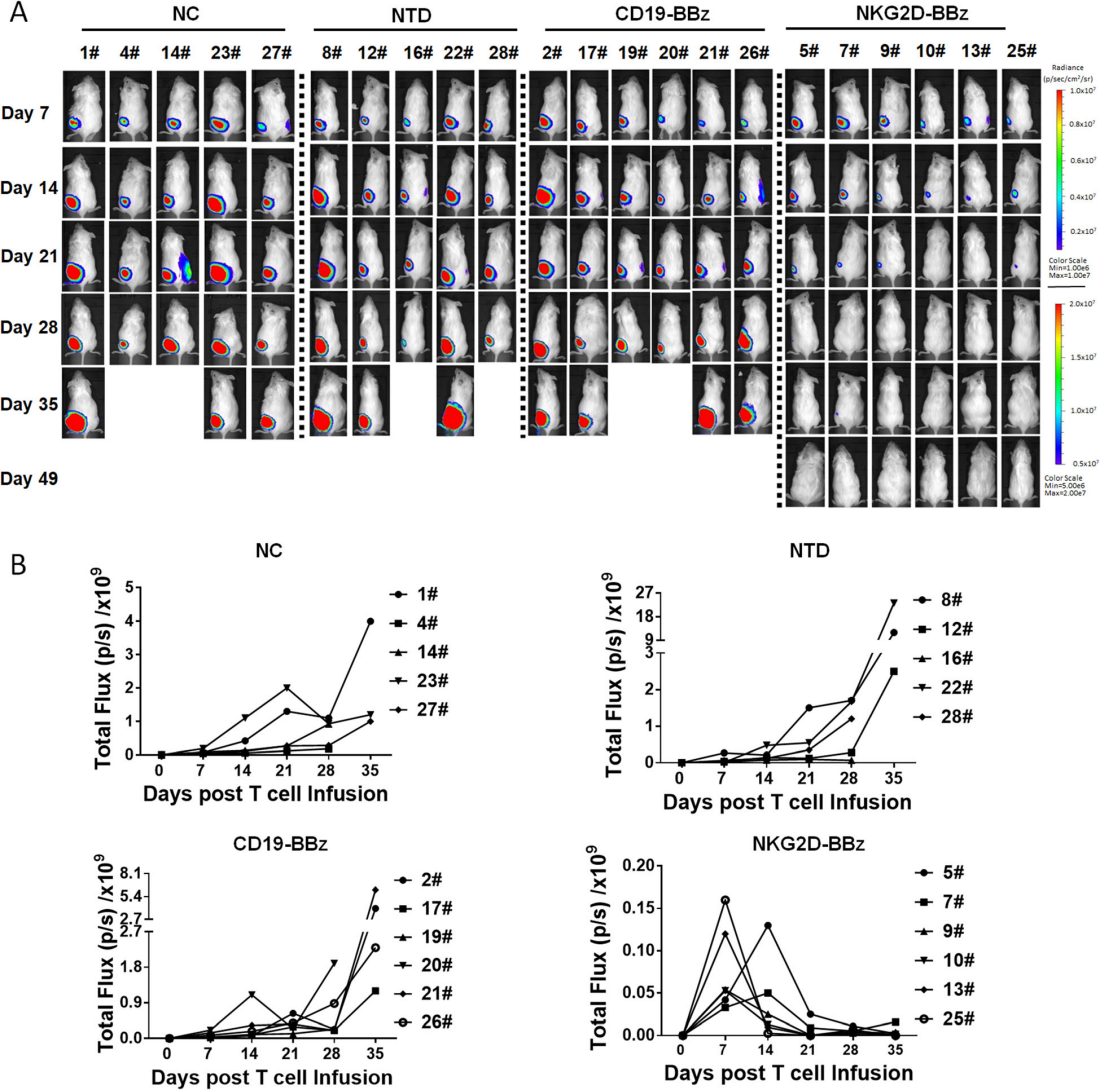
NKG2D-BBz CAR-T cells showed effective and persistent antitumor activity against xenografts formed by U-251MG cells in mice. **a** B-NDG mice were injected with 1 × 10^6^ stable luciferase transfected U-251MG cells subcutaneously and imaged 7 days prior to T cell infusion. After mice received T cells treatment, photographs were taken serially at indicated time. **b** Comparison of tumor bioluminescent signal among the indicated groups at different time points

**Fig. 5:**
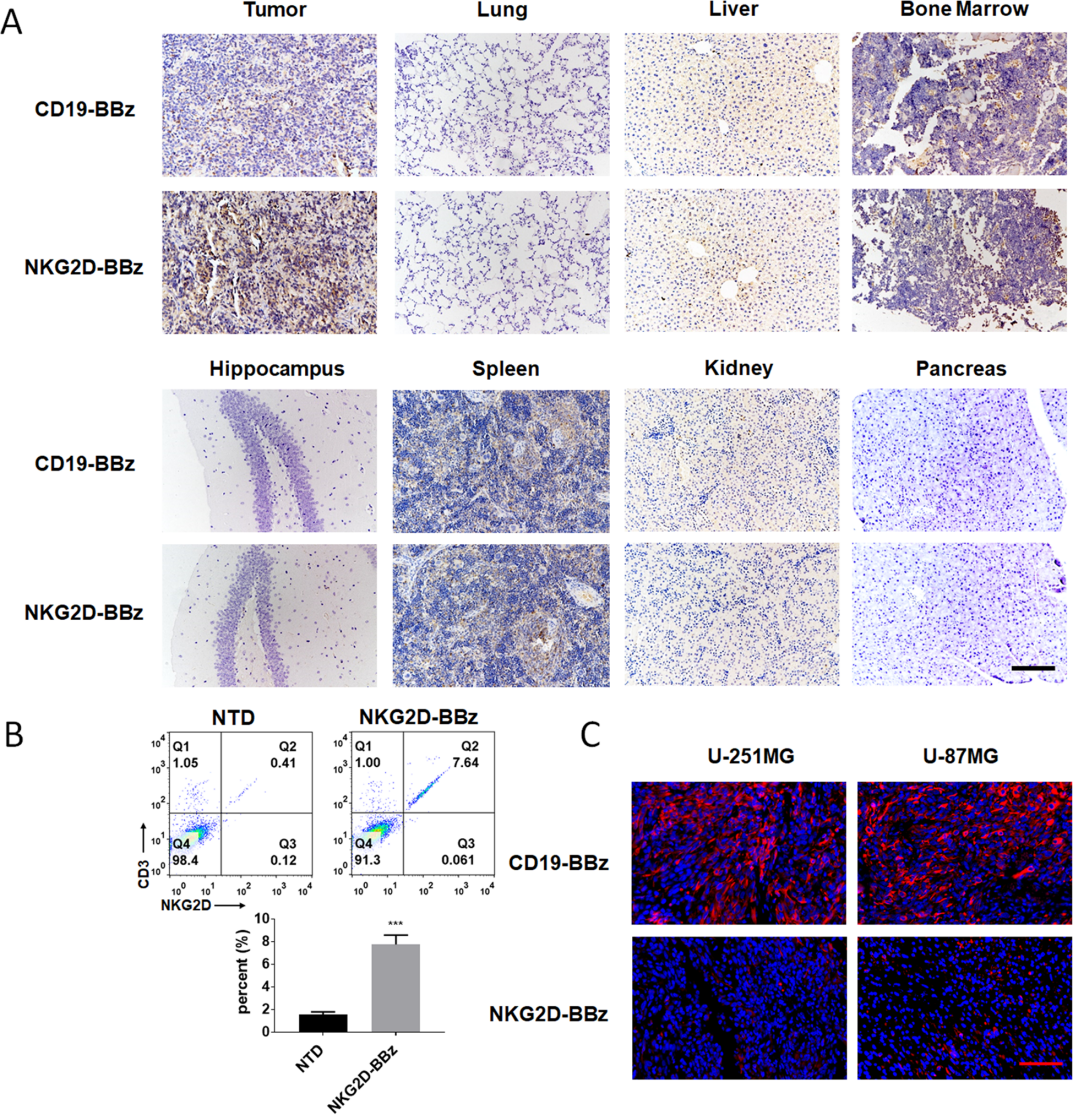
NKG2D-BBz CAR-T cells homed to tumor sites. **a** The mice that received CAR-T cells treatment were sacrificed on day 7 and major organs were collected and immunohistochemical analysis was performed to detect the T cells using a CD3ζ antibody, scale bar = 100 μm. **b** B-NDG mice bearing U-251MG xenografts was sacrificed 7 days after CAR-T cell infusion and the tumor infiltrating lymphocytes (TIL) were analyzed by flow cytometry. The experiment was repeated three times independently. The results are presented as the mean volume ± SD, *** *P* < 0.001. **c** Tumors formed by U-251MG or U-87MG were dissected 7 days after NKG2D CAR-T cell infusion and immunohistochemical analysis was performed to detect the expression of GSC marker NESTIN, scale bar = 50 μm

To evaluate the therapeutic efficacy of NKG2D-BBZ CAR-T cells in vivo, we established subcutaneous xenografts of stable luciferase transfected U-251MG and U-87MG cells in B-NDG mice. When the mean tumor bioluminescence reached ~ 5 × 10^7^ photons/second at one-week after cell injection, mice were administered with normal saline (NS) or NTD, CD19-BBz CAR, or NKG2D-BBz CAR-T cells by intravenous injection. The bioluminescent signals were measured every week until the maximum tumor diameter was approximately 2.5 cm. Compared with the other three groups, the tumors treated with NKG2D-BBz CAR-T cells progressively decreased, and were almost completely diminished at day 21 (Fig. 4 & Additional file 3: Figure S6). No tumor recurrence had been observed till 42-days post-T cell treatment (Fig. 4a & Additional file 3: Figure S6A). The number of CAR-T cells in the treated mice was detected and the results showed that high level of NKG2D BBz CAR- T cells was maintained during the first 24 days, and then the level was gradually decreased (Additional file 3: Figure S7). Immunohistochemical analysis was performed to detect the T cells in the main organs using anti-CD3ζ antibody. The results demonstrated that NKG2D-BBz CAR-T cells accumulated abundantly within the U-251MG cancers, whereas only a few CD19-BBz CAR-T cells were detected within the tumors (Fig. 5). There was no significant difference in the distribution of NKG2D-BBz and CD19-BBz CAR-T cells in other tissues (Fig. 5a). Moreover, we did not find any remarkable lesions in other tested organs of the mice (Fig. 5a). The tumor infiltrating lymphocytes (TIL) were analyzed by flow cytometry, and the results showed that TILs were mainly composed of NKG2D CAR-T cells (Fig. 5b). In addition, we also detected the expression of NESTIN in tumor samples treated with CD19-BBz and NKG2D-BBz CAR- T cells and the results showed that NKG2D-BBz CAR-T cells significantly decreased the percentage of NESTIN-positive cells in tumors. The data suggest that NKG2D-BBz CAR-T cells had cytotoxicity effect targeting glioblastoma stem cells in vivo (Fig. 5c).

### Safety of NKG2D-BBz CAR

**Fig. 6:**
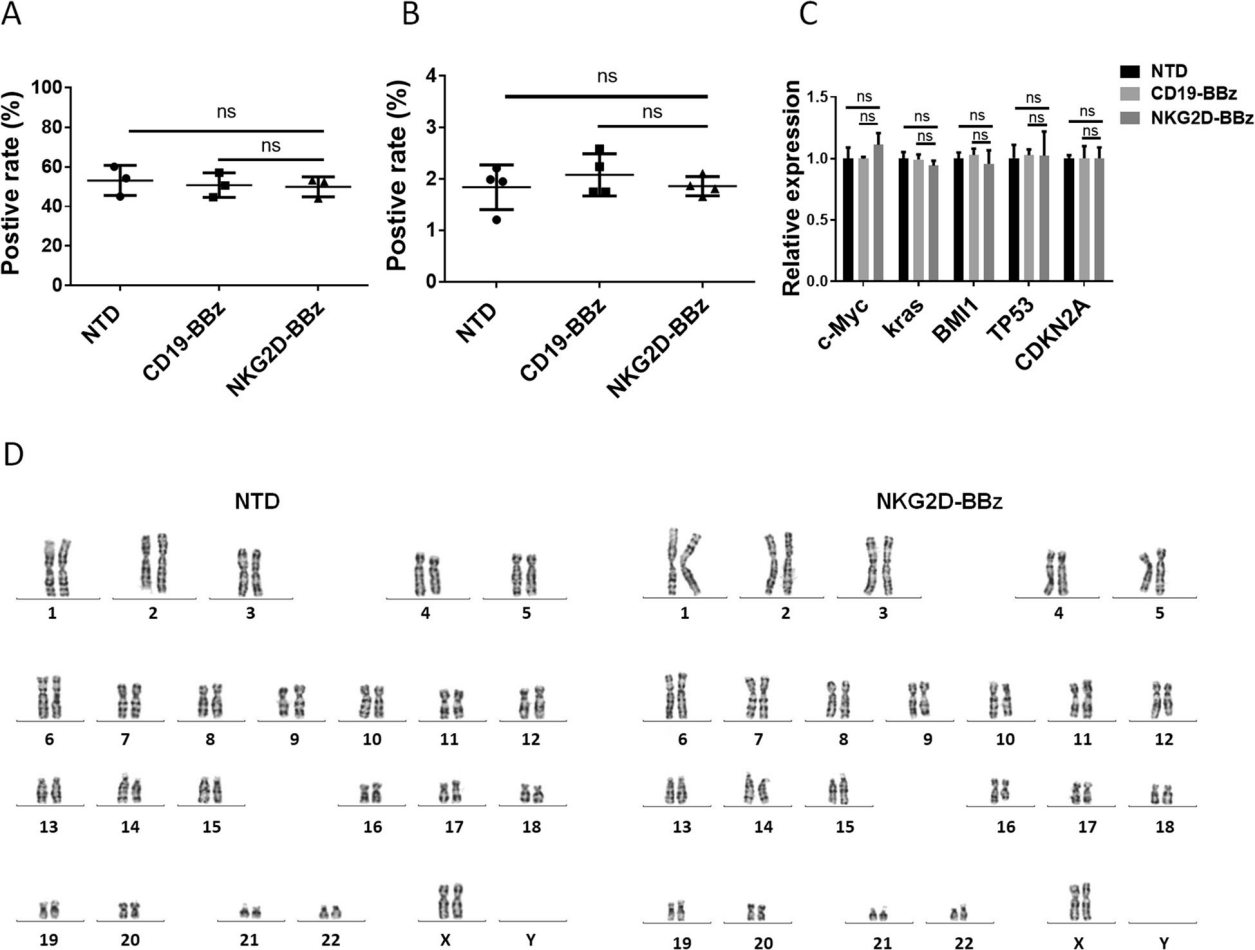
Safety of NKG2D-BBz CAR. **a** Six days after T cell infected by CAR lentivirus, a Click-iT EdU kit was used to detect DNA incorporation. This experiment was repeated three times independently. The results are presented as the mean volume ± SD; ns, not significant. **b** Three days after T cell infected by CAR lentivirus, 7-AAD staining was used to detect dead cells. The results are presented as the mean volume ± SD; ns, not significant. **c** Quantitative RT-PCR analysis for the expression of common oncogenes and tumor suppressor genes in the NTD, CD19-BBz and NKG2D-BBz T cells. The results are presented as the mean volume ± SD; ns, not significant. **d** Karyotype was performed to detect chromosomal alterations in NKG2D-BBz CAR transduction and control untransduced T cells

To analyze the safety of NKG2D-BBZ CAR, the CD19-BBz and NKG2D-BBz CAR transduced and non-transduced T cells were expanded up to 12 days. No significant difference was found in the proliferation rate of these three cell lines (Additional file 3: Figure S8). At the same time, EdU incorporation data indicated that their DNA synthesis rate did not change remarkably (Fig. 6a). Furthermore, apoptosis in the NKG2D-BBz CAR transduced and non-transduced T cells was detected by 7-AAD staining, and all of these data suggested that expression of NKG2D-BBz CAR did not result in obvious cytotoxicity in T cells (Fig. 6b). We also checked the expression of common oncogenes and tumor suppressor genes and found no signs of malignant transformation in the NKG2D-BBZ CAR-T cells (Fig. 6c). Karyotype analysis was performed to analyze the genome instability of NKG2D-BBz CAR-T cells and no abnormal karyotype was found compared with non-transduced T cells (Fig. 6d).

## Discussion

The NKG2D ligands are expressed in a large number of cancer types, including carcinoma of the ovary, colon, lung, breast, kidney, prostate, melanoma, and leukemia [28]. Meanwhile, they are absent or poorly expressed on healthy tissues [13]. Previous studies [29] and our data demonstrated that most gliomas expressed MICA and ULBP proteins, but not MICB. NKG2D-NKG2DL interaction plays an important role in activating the anticancer immune response and has become the focus of immunotherapy. NKG2D-expressing CAR-T cells and NK cells showed effective and persistent antitumor activity against multiple myeloma [30], osteosarcoma [14], ovarian [31], and Ewing sarcoma [32] in the mouse model. In addition, NKG2D CAR-T cells were reported to induce remission in a relapsed acute myeloid leukemia patient [33]. In this study, we demonstrated that the CAR-T cells expressing the extracellular domain of NKG2D lysed human glioblastoma cells and GSCs efficiently in vivo and in vitro.

Till date, EGFRVIII, HER2, and IL-13Rα2-expressing CAR-T cells have been applied in clinical studies of glioblastoma and have shown promising results in a few patients [10]. This demonstrated the feasibility and safety of CAR-T cell-based immunotherapy in glioblastoma treatment. However, there are still many challenges that prevent the further applications of CAR-T in glioblastoma, including inhibition of immune cells by glioblastoma microenvironment, lack of general surface antigen due to high glioblastoma heterogeneity, and limited survival time of CAR-T cells within glioblastomas.NKG2D-expressing CARs have many advantages compared to the existing antigens used in glioblastoma CAR-T cell therapy. 1) NKG2D CAR-T cells can lyse immunosuppressive cells, such as myeloid-derived suppressor cells and regulatory T cells, and induce the immunological response of the host to eliminate immunosuppression caused by tumor microenvironment [31]. Furthermore, the CAR-T cells also target neovasculatures in the tumor microenvironment [34]. 2) NKG2D-expressing CAR-T cells can target multiple tumor-associated ligands. This may prevent cancer immune escape caused by tumor heterogeneity. 3) Previous studies have shown that CAR-T cells expressing the full length murine NKG2D were detected in mouse brain tissues 8 months after inoculation, suggesting that NKG2D CAR-T cells survive much longer than other CAR-T cells in glioblastoma tumors [3, 15]. In our study, the number of NKG2D-expressing CAR-T cell was gradually decreased in the blood along with the reduction of tumor burden. However, a high level of the CAR-T cells was also maintained 45 days post T cell infusion compared with NTD and CD19-BBz groups. It has been reported that many human tumors may evade NKG2D-mediated immune detection by shedding soluble NKG2D ligands from the cell surface. The combination of NKG2D-expressing CAR-T cells with the agents, which mediate inhibition of NKG2D ligands shedding, may overcome the immunosuppressive mechanism. For example, the antibodies, stabilizing MICA and MICB on the surface of tumor cells, were proved to enhance the cytotoxicity of NK cells [35]. In addition, traditional chemotherapeutic agents, such as temozolomide (TMZ) [36], spironolactone (SPIR) [37], gemcitabline [38], and radiotherapy [36], could reinforce the expression of NKG2DLs. Thus, NKG2D CAR-T therapy following traditional treatments could make NKG2D-expressing CAR-T cells more sensitive to tumors.

CSCs were first isolated and confirmed in acute myeloid leukemia (AML) [39, 40] and have been described in most tumors, such as gliomas, lung cancer, liver cancer, colon cancer, and breast cancer [41]. Tumors with a high percentage of CSCs always correlated with a shorter survival time and a poorer prognosis, because of their strong resistance to traditional therapeutics and their ability to restore tumors [42].Therefore, an effective treatment to specifically target CSCs in the tumors is urgently required. Currently, CD133, HER2, and EpCAM-directed CAR-T cells were proved to lyse CSCs in vitro and in vivo [43–45], indicating that the strategy of using CAR-T cells to eliminate CSCs could be a promising treatment for cancers. Here, we confirmed the expression of NKG2DLs in GSCs and demonstrated, for the first time, that NKG2D-expressing CAR-T cells can target GSCs. A previous study has indicated CD133-specific CAR-T cells also killed patient-derived GSCs and prolong the survival of glioma bearing mice slightly [43]. However, in the study the expression of CD57 in CD133-specific CAR-T cells was rapidly up-regulated by direct cell-to-cell contact with CD57-positive target cells, thereby driving the CAR-T cell senescence. Fortunately, no significant change in CD57 expression was found when NKG2D CAR-T cells were incubated with U-251MG cells in our study (data not shown).

We also tested the safety of NKG2D CAR delivered by lentiviral particles, and our results suggested the CAR expression had no significant effect on T cell proliferation, apoptosis, and genomic stability. NKG2DL expression is generally considered to be strictly controlled in healthy tissues to avoid being recognized by the autoimmune system. Although NKG2DL mRNA was detected in healthy tissues, several studies demonstrated post-transcriptional regulation controlled the translation of these proteins [46]. Our data and results from previous studies showed that normal tissues may express low levels of MICA, but the expression is mainly localized in the cell nucleus [46, 47]. Consistent with these results, clinical research on NKG2D-expressing CAR-T cells in acute myeloid leukemia and multiple myeloma did not find any obvious treatment-related safety issues [48]. In our study, the differences in the cytotoxicity of NKG2D-BBz CAR-T cells between U-87MG and the other two cell lines, as well as the differences between U-87MG and U87-CSC, indicated the CAR-T cell cytotoxicity was positively correlated with the cell surface expression of NKG2D ligands. Although U87MG expressed less NKG2DLs, in vivo effect of NKG2D CAR-T cells on it was comparable to U-251MG probably due to the high activity of NKG2D CAR-T cells targeted U87-CSC. Currently, a series of NKG2DLs were reported, including MIC, ULBP in human and H60, Rae-1 and Mult1 in mice. No MIC homologs exist in the mouse and no H60 or Mult1 homologs exist in human. However, ULBP and RAF-1 were identified as equivalents and ULBP1 and ULBP2 were proved to bind to mouse NKG2D [49].The reason for the absence of significant lesions in mice treated with NKG2D CAR-T cells may be the low expression of NKG2DLs in normal tissues or the dramatic difference in NKG2DLs between mice and humans.

## Conclusions

In summary, our results confirmed the high expression of NKG2DLs in human glioblastoma cell lines, cancer stem cells, and tumor samples. Correspondingly, the NKG2D-BBz CAR-T cells efficiently lysed glioblastoma cells and cancer stem cells in vitro and produced high levels of cytokines, perforin and granzyme B. In vivo*,* the CAR-T cells markedly eliminated xenograft tumors and did not exhibit significant treatment-related toxicity in the treated mice. Further, the CAR expression also did not exert any obvious effects on cell proliferation, apoptosis, and genomic stability. These data suggest NKG2D-expressing CAR-T cells may be an encouraging therapeutic approach for glioblastoma patients.

## Additional files

Additional file 1: **Table S1.** List of antibodies used in this study. (XLSX 13 kb)

Additional file 2: **Table S2.** Primers used in this study for real-time PCR. (XLSX 14 kb)

Additional file 3: Figure S1-S8. **Figure S1.** ULBP1 staining in a tissue microarray containing 60 glioblastoma tissues and 10 normal tissues, scale bar = 250 μm. **Figure S2.** ULBP3 staining in a tissue microarray containing 60 glioblastoma tissues and 10 normal tissues, scale bar = 250 μm. **Figure S3.** The cell-surface expression of CD3 in the indicated cells was analyzed by flow cytometry. The RAJI cell line was used as a negative control. **Figure S4.** The morphology of the suspended cell spheres formed in serum-free neural stem cell medium composed of DMEM/F12, 20 ng/ml EGF, 20 ng/ml bFGF, and 1x B27. **Figure S5.** The levels of the indicated cytokines were assessed by ELISA. T cells were incubated with GSC-3# cells at an E:T ratio of 5:1. The results are presented as the mean volume ± SD, ***, *P* < 0.001; ns, not significant. **Figure S6.** NKG2D-BBz CAR-T cells lysed U-87MG cells effectively in mice. (A) B-NDG mice were injected with 1 × 10^6^ stable luciferase transfected U-87MG cells subcutaneously and imaged 7 days prior to T cell infusion. After mice received T cells treatment, photographs were taken serially at indicated time. (B) Comparison of tumor bioluminescent signal among the indicated groups at different time points. **Figure S7.** Persistence of NKG2D-BBz CAR-T cells in mice. B-NDG mice were injected with 1 × 10^6^ stable luciferase transfected U-87MG cells subcutaneously and received T cells treatment 7 days later. Then human genomic DNA in blood was detected using qPCR at indicated time. **Figure S8.** Growth curves for the indicated cells. The CAR-T cells were counted every 2 days. The data are presented as the mean ± SD; ns, not significant. (DOCX 3450 kb)

## Data Availability

The datasets used and/or analyzed during the current study are available from the corresponding author on reasonable request.

## Abbreviations

7-AAD: 7-aminoactinomycin D
B-NDG: NOD-*Prkdc*^*scid*^ *IL2rg*^*tm1*^/Bcgen
CAR: Chimeric antigen receptor
CFSE: Carboxyfluorescein succinimidyl ester
CSCs: Cancer stem cells
DMEM: Dulbecco’s modified Eagle’s medium
DMEM/F12: Dulbecco’s modified Eagle medium: nutrient mixture F-12
EdU: 5-ethynyl-2-deoxyuridine
FBS: Fetal bovine serum
FDA: Food and Drug Administration
GBM: Glioblastoma
MOI: Multiplicity of infection
NKG2D: Natural killer group 2 member D
NKG2DLs: NKG2D ligands
NS: Normal saline
NTD: Non-transduced T cells
PBS: Phosphate buffered saline
RPMI: Roswell Park Memorial Institute
SD: Standard deviation
STR: Short tandem repeat
ULBP: UL16-binding proteins
