# Supplementary material for: T cells expressing NKG2D chimeric antigen receptors efficiently eliminate glioblastoma and cancer stem cells
Source: J Immunother Cancer. 2019 Jul 9;7:171. doi: 10.1186/s40425-019-0642-9 (PMC6617951; doi:10.1186/s40425-019-0642-9)
Supplement: Supplementary file 3 — Figure S1. ULBP1 staining in a tissue microarray containing 60 glioblastoma tissues and 10 normal tissues, scale bar = 250 μm. Figure S2. ULBP3 staining in a tissue microarray containing 60 glioblastoma tissues and 10 normal tissues, scale bar = 250 μm. Figure S3. The cell-surface expression of CD3 in the indicated cells was analyzed by flow cytometry. The RAJI cell line was used as a negative control. Figure S4. The morphology of the suspended cell spheres formed in serum-free neural stem cell medium composed of DMEM/F12, 20 ng/ml EGF, 20 ng/ml bFGF, and 1x B27. Figure S5. The levels of the indicated cytokines were assessed by ELISA. T cells were incubated with GSC-3# cells at an E:T ratio of 5:1. The results are presented as the mean volume ± SD, ***, P < 0.001; ns, not significant. Figure S6. NKG2D-BBz CAR-T cells lysed U-87MG cells effectively in mice. (A) B-NDG mice were injected with 1 × 10^6^ stable luciferase transfected U-87MG cells subcutaneously and imaged 7 days prior to T cell infusion. After mice received T cells treatment, photographs were taken serially at indicated time. (B) Comparison of tumor bioluminescent signal among the indicated groups at different time points. Figure S7. Persistence of NKG2D-BBz CAR-T cells in mice. B-NDG mice were injected with 1 × 10^6^ stable luciferase transfected U-87MG cells subcutaneously and received T cells treatment 7 days later. Then human genomic DNA in blood was detected using qPCR at indicated time. Figure S8. Growth curves for the indicated cells. The CAR-T cells were counted every 2 days. The data are presented as the mean ± SD; ns, not significant. (DOCX 3450 kb) [file 40425_2019_642_MOESM3_ESM.docx]

**Supplementary figures**


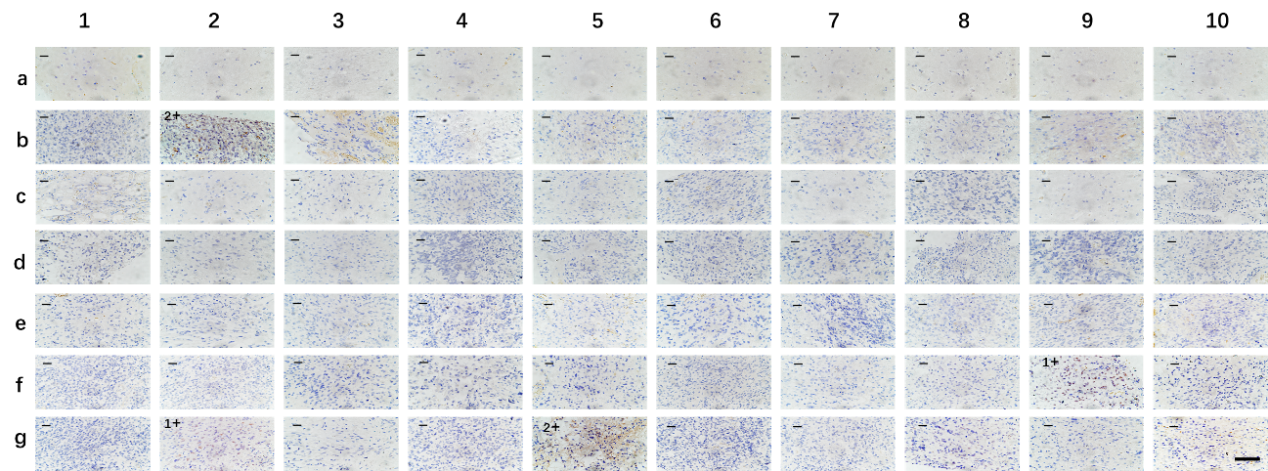
Figure S1

**Figure S1** ULBP1 staining in a tissue microarray containing 60 glioblastoma tissues and 10 normal tissues, scale bar = 250 μm.

Figure S2


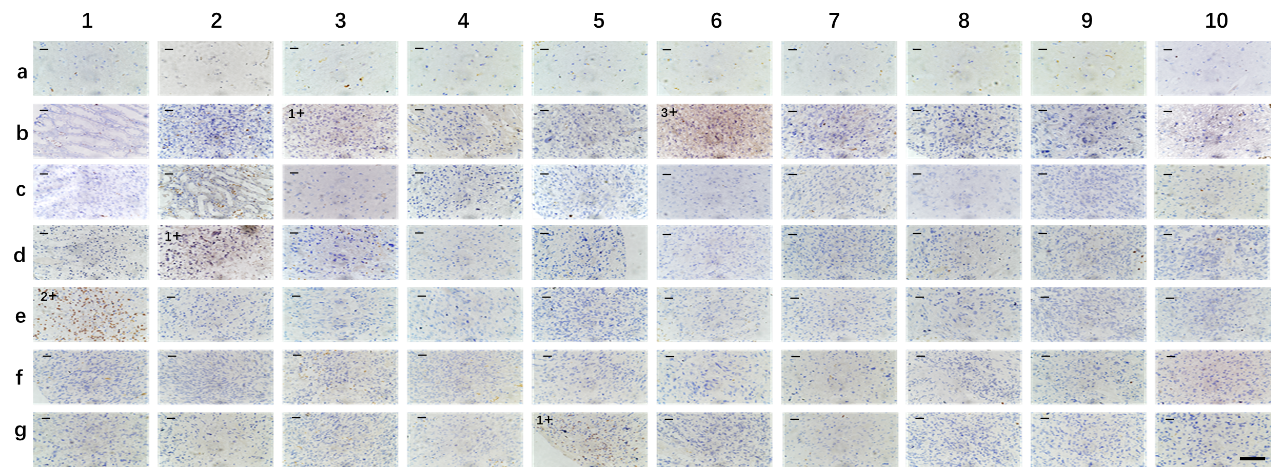


**Figure S2** ULBP3 staining in a tissue microarray containing 60 glioblastoma tissues and 10 normal tissues, scale bar = 250 μm.


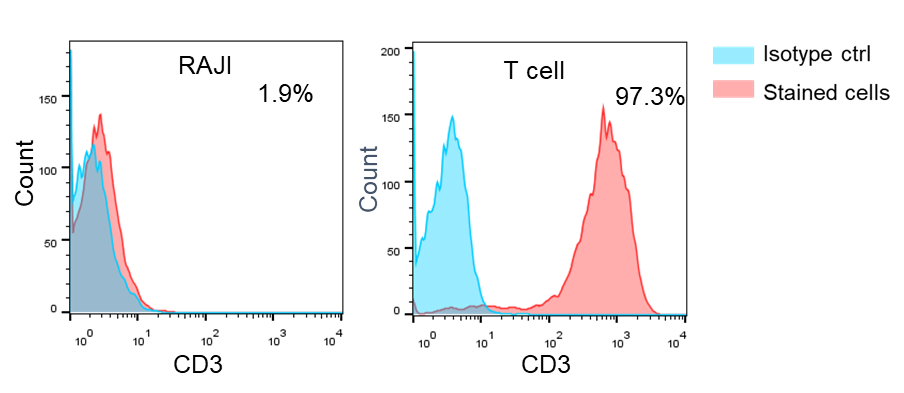
Figure S3

**Figure S3** The cell-surface expression of CD3 in the indicated cells was analyzed by flow cytometry. The RAJI cell line was used as a negative control.

Figure S4


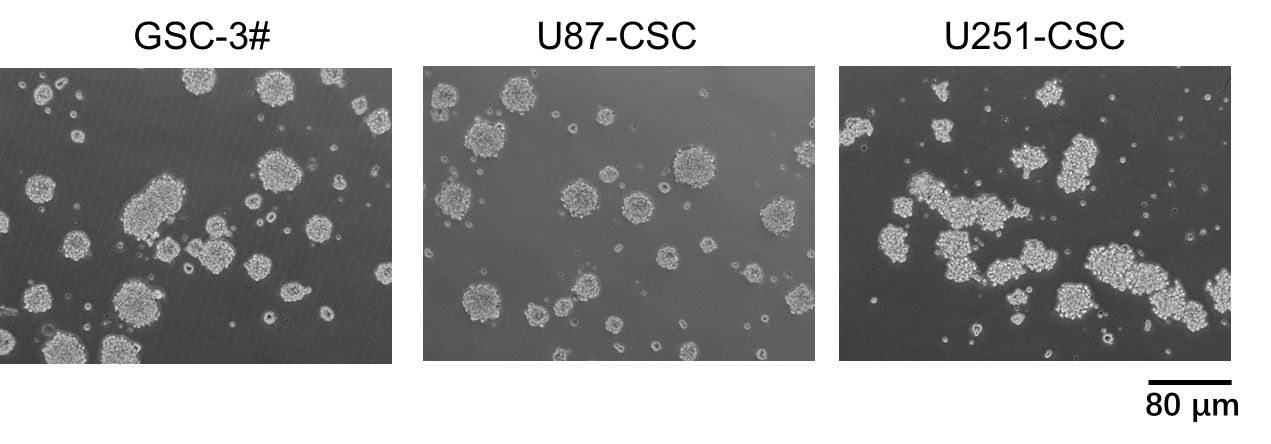


**Figure S4** The morphology of the suspended cell spheres formed in serum-free neural stem cell medium composed of DMEM/F12, 20 ng/ml EGF, 20 ng/ml bFGF, and 1x B27.

Figure S5


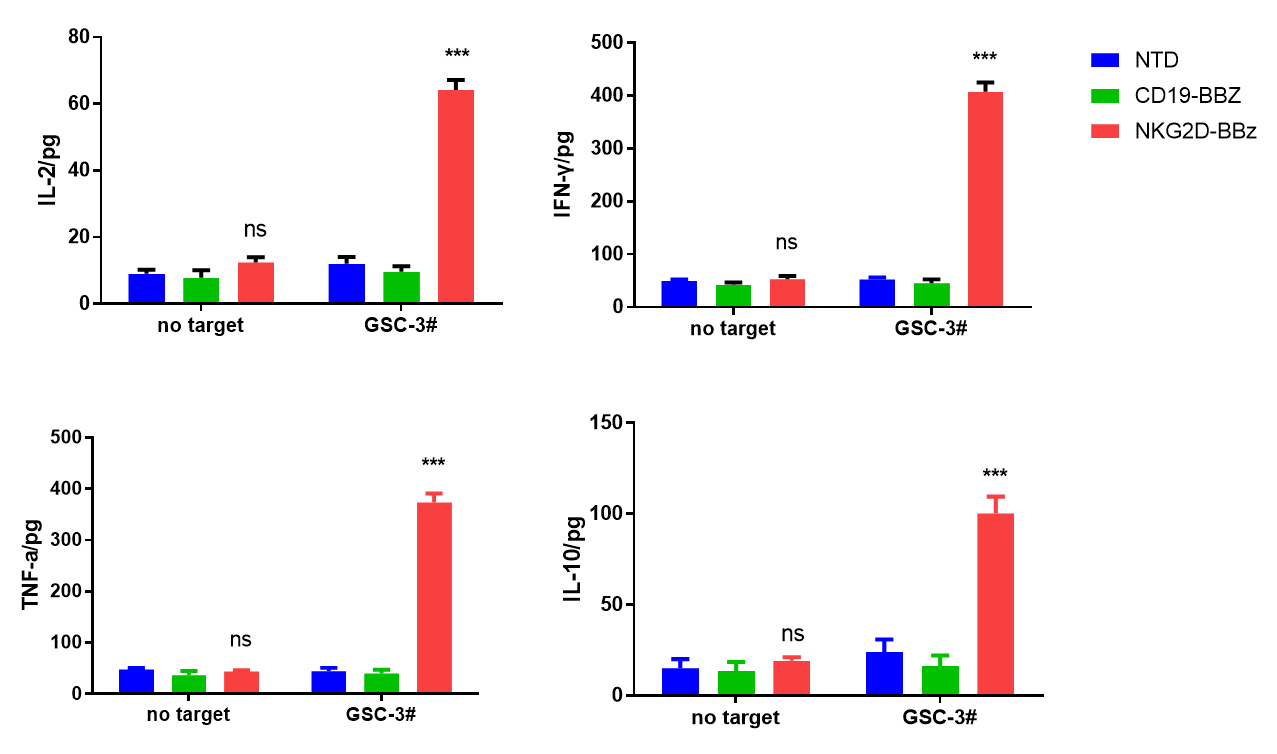


**Figure S5** The levels of the indicated cytokines were assessed by ELISA. T cells were incubated with GSC-3# cells at an E:T ratio of 5:1. The results are presented as the mean volume ± SD, ***, P < 0.001; ns, not significant.


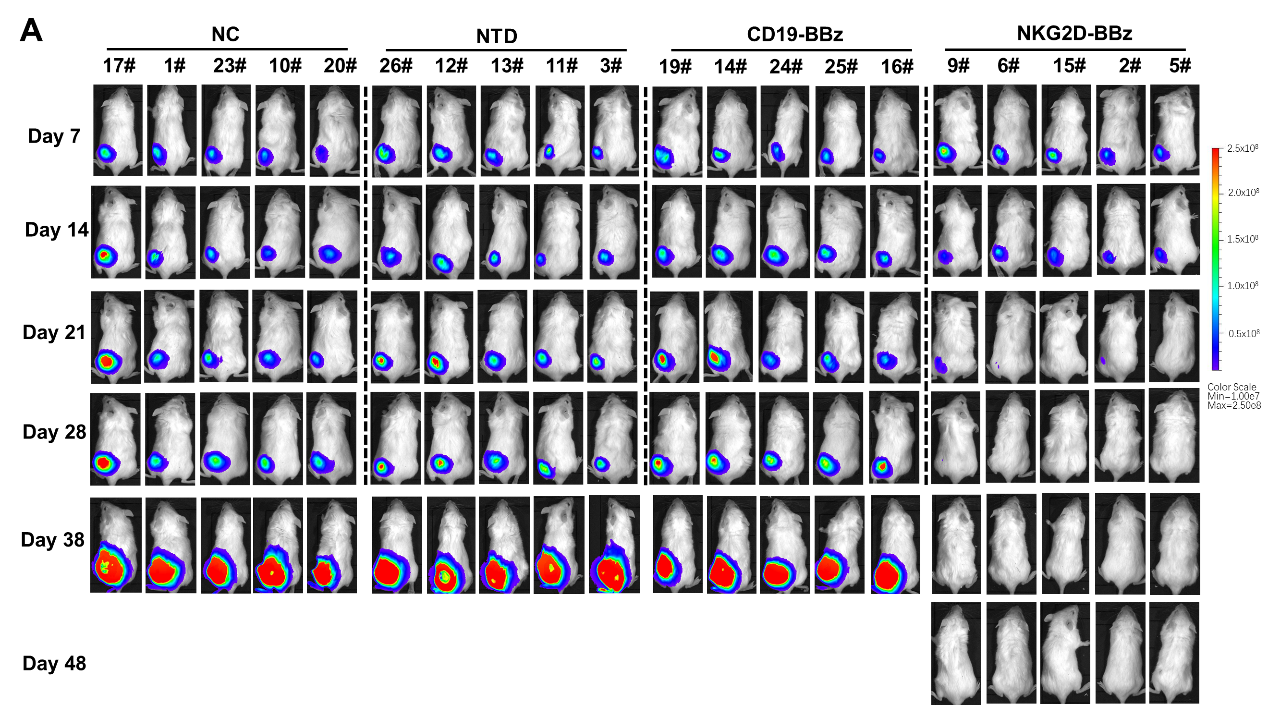
Figure S6


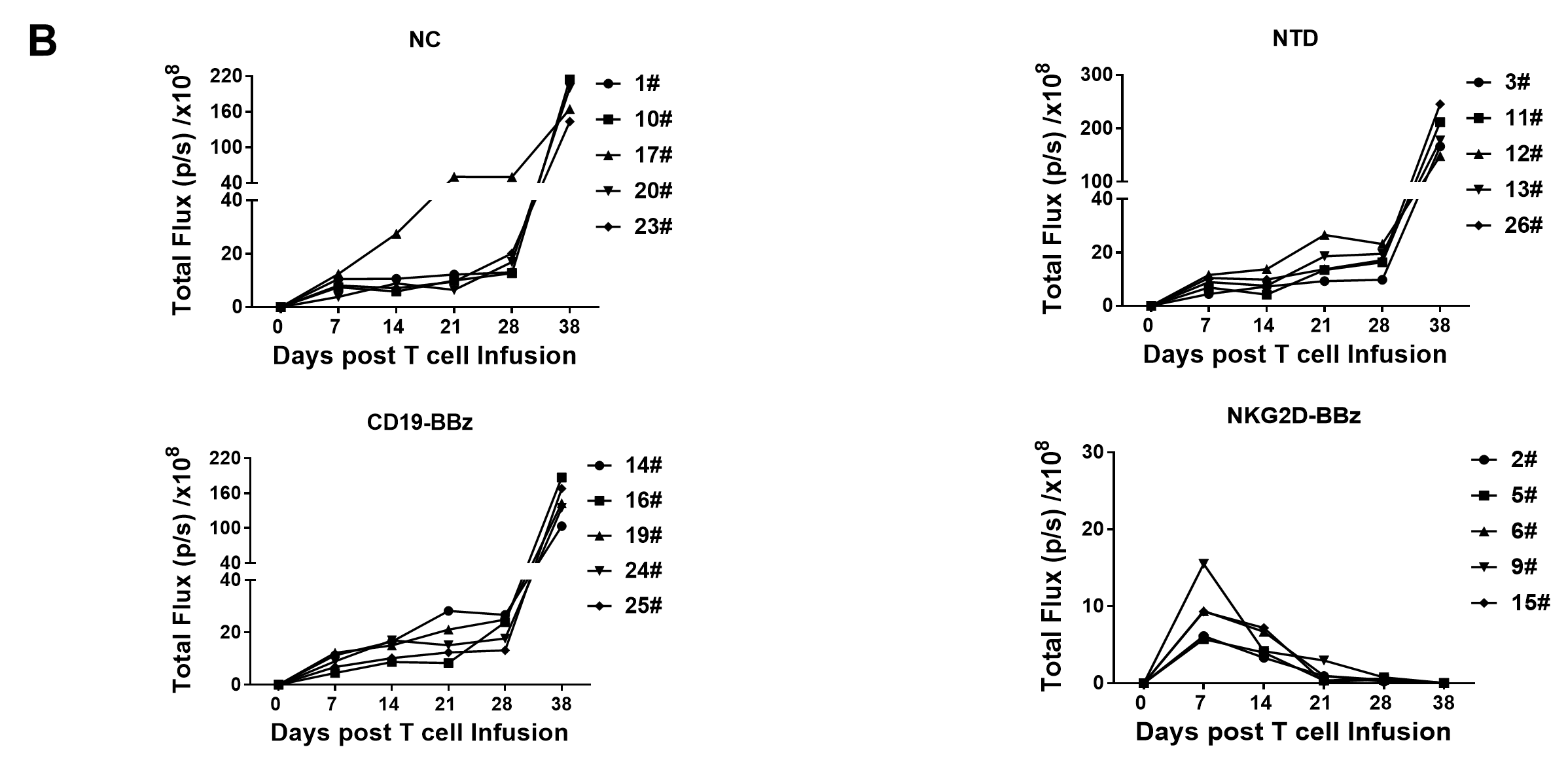


**Figure S6 NKG2D-BBz CAR-T cells lysed U-87MG cells effectively in mice**

(A) B-NDG mice were injected with 1x10^6^ stable luciferase transfected U-87MG cells subcutaneously and imaged 7 days prior to T cell infusion. After mice received T cells treatment, photographs were taken serially at indicated time.

(B) Comparison of tumor bioluminescent signal among the indicated groups at different time points.

Figure S7

**Figure S7** Persistence of NKG2D-BBz CAR-T cells in mice. B-NDG mice were injected with 1x10^6^ stable luciferase transfected U-87MG cells subcutaneously and received T cells treatment 7 days later. Then human genomic DNA in blood was detected using qPCR at indicated time.

Figure S8

**Figure S8** Growth curves for the indicated cells. The CAR-T cells were counted every 2 days. The data are presented as the mean ± SD; ns, not significant.
